# First Insights into the Antiviral Activity of Chitosan-Based Bioactive Polymers towards the Bacteriophage Phi6: Physicochemical Characterization, Inactivation Potential, and Inhibitory Mechanisms

**DOI:** 10.3390/polym14163357

**Published:** 2022-08-17

**Authors:** Olivija Plohl, Katja Fric, Arijana Filipić, Polona Kogovšek, Magda Tušek Žnidarič, Lidija Fras Zemljič

**Affiliations:** 1 Laboratory for Characterization and Processing of Polymers, Faculty of Mechanical Engineering, University of Maribor, Smetanova ulica 17, 2000 Maribor, Slovenia; 2 Department of Biotechnology and Systems Biology, National Institute of Biology, Večna pot 111, 1000 Ljubljana, Slovenia

**Keywords:** bacteriophage phi6, bioactive polysaccharide biomaterials, chitosans, antiviral activity, dynamic and electrokinetic light scattering, charge, biointerfaces

## Abstract

The outbreak of the worrisome coronavirus disease in 2019 has caused great concern among the global public, especially regarding the need for personal protective equipment with applied antiviral agents to reduce the spread and transmission of the virus. Thus, in our research, chitosan-based bioactive polymers as potential antiviral agents were first evaluated as colloidal macromolecular solutions by elemental analysis and charge. Three different types of low and high molecular weight chitosan (LMW Ch, HMW Ch) and a LMW Ch derivative, i.e., quaternary chitosan (quart-LMW Ch), were used. To explore their antiviral activity for subsequent use in the form of coatings, the macromolecular Chs dispersions were incubated with the model virus phi6 (surrogate for SARS-CoV-2), and the success of virus inactivation was determined. Inactivation of phi6 with some chitosan-based compounds was very successful (>6 log), and the mechanisms behind this were explored. The changes in viral morphology after incubation were observed and the changes in infrared bands position were determined. In addition, dynamic and electrophoretic light scattering studies were performed to better understand the interaction between Chs and phi6. The results allowed us to better understand the antiviral mode of action of Chs agents as a function of their physicochemical properties.

## 1. Introduction

The outbreak of severe acute respiratory syndrome coronavirus 2 (SARS-CoV-2), which led to more than 569 M infections (assessed on 10 August 2022, https://www.worldometers.info/coronavirus/), and a death record of 6.4 M, has revealed many shortcomings, including inadequate or less adequate protective equipment, which is seen as a priority pillar to strengthen the foundation of preventive care. To improve protection and combat the COVID-19 epidemic, and other respiratory virus diseases, progress needs to be made in the availability and antiviral efficiency of personal protective equipment (PPE), which includes face masks, protective suits, overalls, and gloves [1,2]. It has been shown that one of the mechanisms of enveloped virus transmission, such as SARS-CoV-2, is through the surface of various materials [3,4]. Therefore, by engineering the material’s surface, the incidence of contamination with viruses and other micro-organisms can be reduced [5]. A possible and efficient way to combat epidemics is to functionalize the surface of personal protective materials and devices with antiviral compounds in the form of coatings [5,6,7]. Due to the great surface area and thus accessibility of the functional surface groups, numerous synthetic, inorganic, and photocatalytic (nano)coatings have been proposed to efficiently inactivate enveloped viruses, for example SARS-CoV-2. However, several drawbacks, such as limited efficacy, environmental contamination, and toxicity make them less attractive and call for alternative solutions [7,8]. An approach to overcome these drawbacks could be the development of coatings based on natural substances, such as biopolymers. Biopolymers, especially polysaccharides, are abundant, biodegradable, renewable, biocompatible, and some of them (such as chitosan and its derivatives, alginates and carrageenan) show potential antiviral properties [9,10,11,12]. Great attention has also been given to the development of drugs and vaccines based on polysaccharides for use against SARS-CoV-2 [10,11].

Among polysaccharides, chitosan (Ch) (an amino group-based polysaccharide formed by deacetylation of chitin [13]) and its derivatives have been extensively studied for their antimicrobial properties in various segments, such as food packaging [14,15,16], bio-medical applications [17,18,19,20], and technical applications, including heavy metal removal [21,22]. Various properties of Ch and its derivatives (e.g., concentration, degree of polymerization, degree of deacetylation, positive charge, MW, and chemical modification) have been shown to play a crucial role in viral suppression [23]. Unfortunately, the antiviral activity of pure Ch has been shown to be limited due to its poor solubility at a pH above the isoelectric point (IEP), and this is why its derivatization is needed.

Chitosan-based compounds have demonstrated good antiviral activity against many human, animal, plant, and bacterial viruses (bacteriophages) (reviewed by [23,24]). Chitosan derivatives showed inhibitory effects against bacteriophages, such as T2, T7 [25], 1-97A [26], MS2 [27,28], and phiX17 [28]; animal viruses including murine norovirus [29], feline calicivirus [27,28], Newcastle virus [30]; and plant viruses, e.g., the tobacco mosaic virus [31]. However, understanding of the potential antiviral activity of Chs against representative human enveloped respiratory viruses and their surrogates, such as bacteriophage phi6, is still lacking.

Recently, special attention was given to studies of chitosan activity against SARS-CoV-2 [11,24,32,33]. The cationically modified chitosan derivative N-(2-hydroxypropyl)-3-trimethylammonium chitosan chloride (HTCC) and hydrophobically modified HTCC have been shown to be potent inhibitors of human coronavirus NL36 replication [34] and to encourage inhibition of the virus–receptor interaction [35]. HTCC has therefore been proposed as highly potent polymeric inhibitors of SARS-CoV-2 and MERS-CoV [36], but none of these studies have examined the physicochemical properties of the chitosan-based agent in relation to its antiviral properties.

To understand the effect of chitosan-modified solid surfaces, it is first necessary to investigate how the physicochemical properties of Ch in the form of polymeric macromolecular solutions or particle dispersions affect the virus. In the present study, we evaluated the effect of physicochemical properties of three types of chitosan-based polymers (low molecular weight chitosan, LMW Ch; high molecular weight chitosan, HMW Ch; and Ch derivative, i.e., quaternary low molecular weight chitosan, quart-LMW Ch) as colloidal macromolecular formulations on viruses. We used dsRNA bacteriophage phi6, which is widely accepted as a model surrogate for SARS-CoV-2 due its similar size and the presence of the lipid envelope containing spike proteins [37]. To characterize Ch, an elemental analysis with an ATR-FTIR and X-ray photoelectron spectroscopy (XPS) were performed and their charge was determined, with potentiometric and polyelectrolyte titrations. Infectivity assays were used to determine inactivation potential, i.e., the antiviral activity of Chs, whereas the change in the virus morphology after its interaction with Chs was observed using transmission electron microscopy (TEM). Changes in infrared bands positions after contact between the chitosan and phi6 were inspected with attenuated total reflectance Fourier transform infrared spectroscopy (ATR-FTIR). Furthermore, dynamic and electrophoretic light scattering studies by means of zeta potential (ZP) and hydrodynamic diameter (HD) were performed either for Chs or phi6, alone or after their interaction. The results provide a better understanding of the Ch antiviral mode of action as a function of their physicochemical properties. This report is thus the first that explains the interaction and inactivation of bacteriophage phi6 with chitosan-based compounds, where surface charge was proven to be the key element for phi6 inhibition. Additionally, the methods and approach adopted in this paper propose a methodology in which chemistry (surface colloid chemistry) interventions provide an additional view of the interaction phenomena between biopolymer and phi6 in combination with in vitro microbiological assays. The obtained results will further contribute to the understanding and application of Ch and its derivatives as an antiviral coating against SARS-CoV-2 on various surfaces.

## 2. Materials and Methods

### 2.1. Materials

Low molecular weight chitosan (LMW Ch: 50,000–190,000 Da, 75–85% deacetylated), high molecular weight chitosan (HMW Ch; 310,000–375,000 Da, >75% deacetylated), glacial acetic acid (AcOH: 99–100%), and glycidyltrimethylammonium chloride GTMAC (technical, >90%) were supplied by Sigma-Aldrich, Vienna, Austria. Milli-Q/ultrapure water (resistivity of 18.2 MΩ cm at 25 °C) was prepared using the Milli-Q system (Millipore Corporation, Bedford, MA, USA). NaOH (˃98%) was purchased from Honeywell (Seelze, Germany).

The chemicals used for the double-layer plaque assay included trypticase soy broth (BD) (TSB) (from Sparks, NV, USA) Bacto agar (BD) (from Sparks, USA), MgCl_2_·6H_2_O (Duchefa Biochemie, Haarlem, The Netherlands), and ampicillin sodium salt (Sigma-Aldrich, Spruce Street, St.Louis, MO, USA).

### 2.2. Preparation of LMW and HMW Chs Colloidal Macromolecular Formulation

Aqueous LMW and HMW Chs solutions at 2% (*w*/*v*) were prepared by dissolving HMW and LMW Ch powders in MilliQ water. Glacial acetic acid was added dropwise to the Chs solution with continuous mechanical stirring to allow dissolution of the Chs. The solutions were stirred for 24 h until a homogeneous macromolecular dispersion was obtained. The pH was adjusted to 4.0. For further analysis, the HMW and LMW Chs dispersions prepared were diluted to the target concentration and the pH was adjusted to 4 for further experiments.

### 2.3. Quart-LMW Ch Preparation

**Scheme 1:**
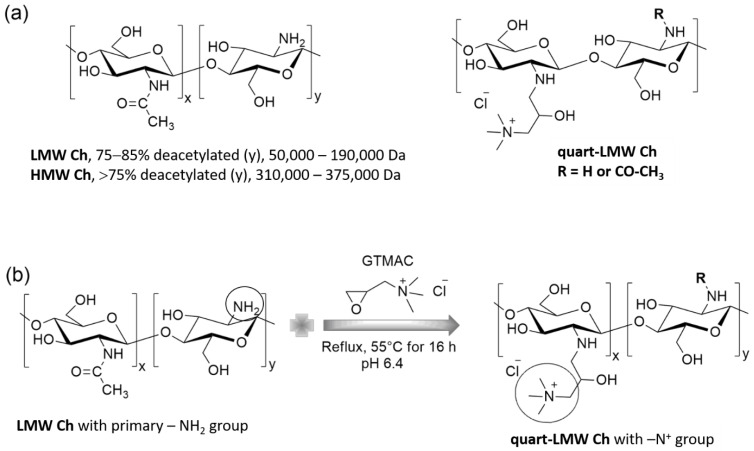
(**a**) Scheme of HMW, LMW, and quart-LMW Chs and (**b**) derivation procedure of LMW Ch to quart-LMW Ch.

Quaternary LMW Ch was prepared by derivatization of LMW Ch with the reagent GOPTS, where the primary amino groups react with the epoxide ring of GOPTS to form a secondary amine with a quaternary backbone (Scheme 1). The modification of LMW Ch was carried out in accordance with previously published references [38,39] with some minor modifications, namely a different time of LMW Ch mixture preparation. Briefly, 2.5 g of LMW Ch was dispersed in 100 mL of MiliQ water, and 0.5% acetic acid (10 mL) was slowly added to the prepared mixture and stirred for 24 h. The pH of the mixture was 6.4 after the addition of acetic acid and remained the same after 24 h of mixing with the magnetic stirrer. Following this, 6.9 mL of GTMAC reagent was slowly added to the LMW Ch mixture and refluxed at 55 °C for 18 h, with continuous magnetic stirring. After the derivatization process, the obtained mixture was cooled and the pH of the suspension was increased to 8.5 after the reaction. Centrifugation was then carried out at 4000 rpm for 20 min to obtain only the derivative quart-LMW Ch, while the unreacted LMW Ch settled. After centrifugation, the supernatant was poured into 150 mL of acetone and the mixture was centrifuged again at 4000 rpm for 20 min. The supernatant was removed while the sediment was pre-dried and re-dissolved in water, precipitated with an excess of acetone, and centrifuged again. This procedure was repeated twice, and the final precipitated product was dried in a heater.

### 2.4. Chitosan-Based Polymers Characterization

#### 2.4.1. Attenuated Total Reflectance Fourier Transform Infrared Spectroscopy (ATR-FTIR)

Infrared spectra of lyophilized HMW, LMW, and quart-LMW Chs were recorded using a Pelkin Elmer Spectrum GX NIR FT-Raman spectrometer (Waltham, MA, USA) containing a diamond crystal ATR accessory in transmission mode. The background spectra and then the sample spectra were recorded with 32 scans at a resolution of 4 cm^−1^ in the wavenumber range (4000–400 cm^−1^). All the spectra obtained were deconvoluted using automatic smoothing filters, automatic baseline corrections, and ATR corrections, and finally normalized to 1. All measurements were conducted in ambient conditions (temperature = 25 °C, relative humidity = 55%).

#### 2.4.2. X-ray Photoelectron Microscopy (XPS)

The survey spectra of HMW, LMW, and quart-LMW Ch, as well as the high-resolution spectra of C 1s, O 1s, and N 1s, were obtained using an XPS instrument PHI-TFA 5600 XPS spectrometer from Physical Electronics Inc. (Chanhassen, MN, USA). A base pressure of approximately 6 × 10^−8^ Pa prevailed in the instrument chamber. All samples were excited with X-rays over the 400 µm spot area, using monochromatic Al Kα1.2 radiation (1486.6 eV) at 200 W in at least 2 different locations of each sample. The photoelectrons produced were detected using a hemispherical analyzer. The latter was positioned at an angle of 45 ° to the sample surface. An additional electron gun was used to compensate for the possible charging effect of the samples. The energy resolution was ~0.6 eV. The concentrations of the surface elements were calculated from the spectra of the survey scans using Multipak software, version 9.6.0. A high-resolution C 1s spectrum with a binding energy of 284.8 eV was used to correct the binding energy scale.

#### 2.4.3. Potentiometric and Polyelectrolyte Titration

Potentiometric titration of all Chs was performed to determine the total charge in mmol/g and p*K* values. All solutions required for potentiometric titration were prepared using ultrapure water with low carbonate content (<10^−6^ M). The pH-dependent titration was carried out at pH values ranging from 2 to 11 using 0.1 M HCl and 0.1 M KOH aqueous solutions as titrants. For this purpose, the combined glass electrode (InLab Reach 225, Mettler Toledo, Greifensee, Switzerland) was equipped with a double burette titrator (T-70, Mettler Toledo, Greifensee, Switzerland). Before starting titration, the individual ionic strength of the macromolecular dispersion was adjusted to 0.1 M with 3 M KCl. The ionic strength therefore remained within 2% of the initial value when 0.1 M KOH and HCl were added. The blank HCl-KOH titration was performed in the same manner as described above. A more detailed description of the charge calculations and the p*K* value determination can be found elsewhere [40].

The degree of quaternization of quart-LMW Ch was determined by polyelectrolyte titration based on the ionization group differences between positively charged primary and quartenized amino groups at pH 2.2, and for only quartenized groups at pH 8. The titrant used in the polyelectrolyte titration was negatively charged polyelectrolyte polyethylene sulfonic acid sodium salt (PES-Na). The cationic indicator toluidine blue (TB) was used to determine the equivalence point photometrically. A Mettler Toledo DL 53 titrator with a 10 mL burette was used for the stepwise addition (0.1 mL every 3–10 s) of PES-Na (c = 0.01 M) to 30 mL of the polymeric macromolecular dispersion. Absorbance was measured as a potential change in mV using a Mettler Toledo DP5 phototrode at a wavelength of 660 nm. The amount of positively charged groups at 2 different pH values can be estimated from the volume of titrant in solution at the equivalence point, by assuming a 1:1 binding stoichiometry between positively charged amino and quaternized groups of Ch product and sulphonated groups of PES-Na.

### 2.5. Determination of Antiviral Activity of Chitosans

#### 2.5.1. Infectivity Assays

Antiviral activity of the chitosans was determined using an enveloped bacteriophage phi6 (DSM 21518). Bacteriophage suspensions in a 1 × SM buffer at concentrations of ~10^6^ plaque-forming units (PFU)/mL were added 1:1 to the suspension of test biopolymer compounds (LMW, quart-LMW, or HMW Chs). The final concentration of the test compounds was 1.25 mg/mL, which were chosen based on their minimum inhibitory concentrations on bacteria, which phi6 infect (Supplementary Material, Tables S1 and S2). The suspension was then thoroughly mixed and incubated at room temperature for 2 h. In 1 sample, the test compound was replaced with a 1 × SM buffer, and this sample served as a positive control (PC) for the infectivity tests, i.e., double-layer plaque assays (DAL). Experiments were performed at either higher (7.4 ± 0.4) or lower (4.5 ± 0.3) pH in order to evaluate chitosan protonation behaviour on antiviral activity. After the incubation period, the appropriate dilutions of the samples were prepared, and virus concentrations were determined using a DAL as described below.

##### Double-Layer Plaque Assay (DAL)

To determine virus inactivation using a DAL, 3 medias were prepared: ‘TSB agar’, ‘TSB top agar’, and ‘liquid TSB’. ‘TSB agar’ was used to prepare agar plates from 30 g/L TSB and 15 g/L agar. ‘TSB top agar’, used for the top layer of the double-layer plaque assay, was prepared in the same way as ‘TSB agar’, except that 7 g of agar was added. ‘Liquid TSB’ was used for culturing bacterial cultures *Pseudomonas syringae* van Hall 1902 (DSM 21482) for phi6 tests. All media contained 1.93 g/L MgCl_2_·6H_2_O.

The DAL was performed with a bacterial host culture in the logarithmic phase, prepared by inoculating 0.2 mL of a 2-day old (for phi6) bacterial culture in 5 mL of liquid TSB. This was followed by ≥ 3 h incubation at 25 °C and 210 rpm for phi6. The DAL was performed by adding 0.2 mL (for phi6) of the bacterial host and 0.25 mL of undiluted or diluted samples (virus samples with test agents or PC) to ~5 mL of melted ‘TSB top agar’ in 15 mL glass tubes. This was then thoroughly mixed and poured onto agar plates. After overnight incubation at 25 °C (for phi6), the number of plaques were counted and the virus concentration was calculated by considering virus dilutions and plating volumes as follows:

(1) $$Inacitvation\;\left(\%\right)=\frac{A-B}{A}\times 100\;\;$$

(2) Inacitvation (log)=log(A)−log(B)

where *A* is the virus concentration in PC after 2 h incubation and *B* is the virus concentration in samples with antiviral agents after 2 h incubation. Both concentrations were calculated as the average of 1 or 2 dilutions, with 2 or 3 technical replicates.

### 2.6. Interaction of Chitosans with Phi6 and Mechanisms of Inactivation

#### 2.6.1. Observation of Virus Morphology with Transmission Electron Microscopy (TEM) 

The morphology of bacteriophage phi6 with or without test agents was visualized with TEM, using a negative staining method. Due to the lower sensitivity of TEM, we used higher initial concentrations of phi6 (~1 × 10^11^ PFU/mL) than in infectivity assays. Test agents (LMW Ch, quart-LMW Ch, or HMW Ch) were mixed 1:1 with bacteriophage phi6 in a 1 × SM buffer without gelatin. The final concentration of the test agents was 1.25 mg/mL. In the positive control, the test agent was replaced with the same buffer. The suspensions were then thoroughly mixed and incubated at room temperature for 2 h. Samples were applied onto freshly glow-discharged 400-mesh copper grids, incubated for 5 min, washed with few droplets of deionized water and stained with 1% (*w*/*v*) water solution of uranyl-acetate (SPI, USA). The grids were observed with a TEM TALOS L120C (Thermo Fischer Scientific, Waltham, MA, USA) operating at 100 kV, and micrographs were captured by CCD camera Ceta 16M using Velox software for micrographs acquisition and processing. 

#### 2.6.2. ATR-FTIR of Antiviral Agent-Model Virus

For exemplary chosen ATR-FTIR analysis of phi6, LMW Ch, and phi6 + LMW Ch, all samples were air dried and lyophilized. The stock solution of phi6 was first dried and then lyophilized to remove excess water. A control sample of the components was contained in the solution without phi6 (SM buffer, etc.). Its IR spectrum was also measured and subtracted from the actual phi6 phage spectrum. LMW Ch was analyzed as a previously prepared macromolecular dispersion and then dried in a dryer and finally lyophilized. The combination of phi6 + LMW Ch was prepared in the same way as the infectivity assays (Section 2.5.1). Both were incubated for 2 h. The mixture of LMW Ch + phi6 was then ultra-centrifuged with an Eppendorf miniSpin centrifuge at 15,000 rpm for 1 min and then air dried, followed by freeze drying. The ATR-FTIR for all samples was performed as already explained in Section 2.4.1, using a Pelkin Elmer Spectrum GX NIR FT-Raman spectrometer (Waltham, MA; USA) containing a diamond crystal ATR accessory in ambient conditions (temperature = 25 °C, relative humidity = 55%) in transmission mode.

#### 2.6.3. Dynamic Light Scattering and Electrophoresis Experiments in Antiviral Agent-Model Virus Suspension

Dynamic light scattering (DLS) to measure the mean hydrodynamic diameter of each component was performed on the Anton Paar LiteSizer 500 instrument using the Omega cuvette. We tested LMW, HMW, and quart-LMW Chs at 1.25 mg/mL and bacteriophage phi6 (concentration at 10^6^ PFU/mL), individually or as a combination of bacteriophage with each Ch. These combinations were prepared as described in Section 2.5.1 and had a transmittance of approximately 90%, meaning that enough light is transmitted through them to obtain a reliable result. Prior to measurement, the same volume of Chs antiviral agent was mixed with phi6 to achieve a final concentration of 1.25 mg/mL of Chs (in the same way as discussed in 2.5.1), and the pH was then adjusted to 7.4 or 4.5 with 0.1 M NaOH or dilute acetic acid, respectively. The incubation time was 2 h at room temperature. Subsequently, all samples were analyzed in their native form without filtration to obtain a clear picture of the inactivation activity. Measurements were performed at 25 °C, at 175 ° measurement angle, and in automatic quality mode. The peak particle diameter (nm) and polydispersity index (PDI) for the different samples were determined. The LMW, HMW, and quart-LMW Chs were measured in the standard polystyrene latex mode, while the bacteriophage and mixtures were measured in the protein mode, which introduces small pauses between measurement runs and therefore limits the Joule heating. For each sample, 3 replicates were performed, and the results were averaged. The data were collected using Anton Paar’s Kaliope software.

In general, the zeta potential is closely related to the surface charge of particles and provides an indication of the stability of colloidal systems. It is strongly influenced by pH and also by ionic strength. The zeta potential can be calculated using Henry’s equation [41]. The measurement of zeta potential by electrophoretic light scattering (ELS) of the same samples, as in the case of the hydrodynamic diameter, was performed on a Litesizer 500 instrument at 25 °C in an Omega cuvette. The voltage was set automatically by the instrument. The quality was set to automatic mode, with 200 runs per measurement. The optical filter and focus position were automatically selected by the instrument. For each sample, 3 replicates were performed, and the results were averaged.

## 3. Results and Discussion

### 3.1. Physicochemical Characterization of Chitosan-Based Polymers as Potential Antiviral Agents

#### 3.1.1. ATR-FTIR of Chitosans

**Figure 1:**
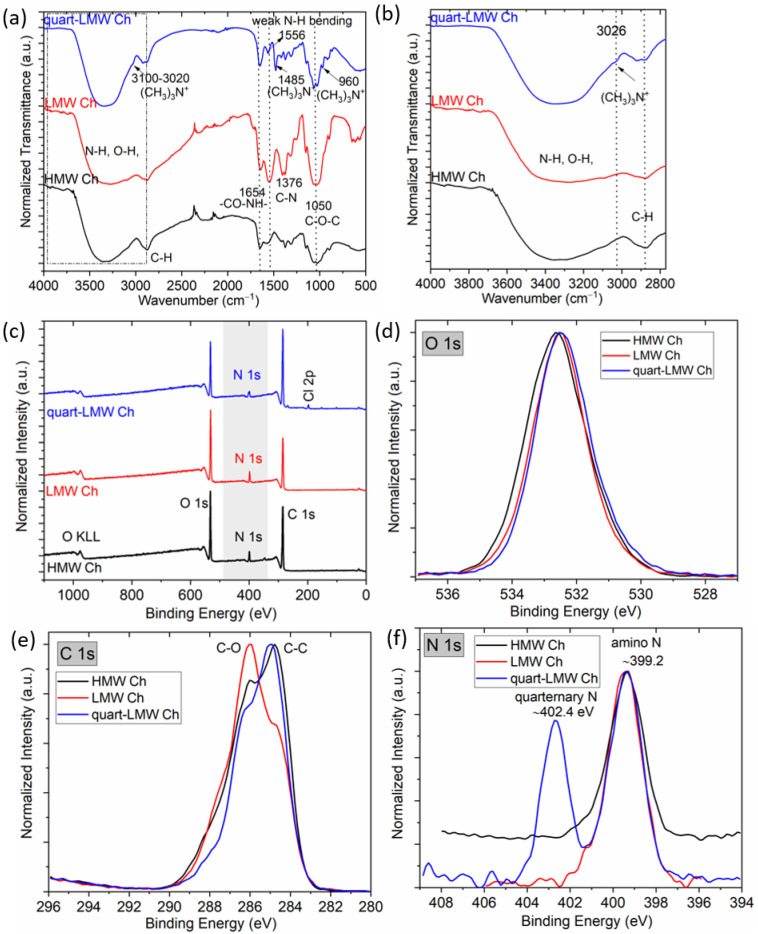
(**a**) ATR-FTIR spectra for LMW, HMW, and quart-LMW Chs with the most important bands marked, (**b**) the magnified portion of IR spectra at larger wavenumbers, (**c**) survey spectrum for HMW, LMW, and quart-LMW Chs, and, corresponding high-resolution spectra for (**d**) O 1s, (**e**) C 1s, and (**f**) N 1s.

**Figure 2:**
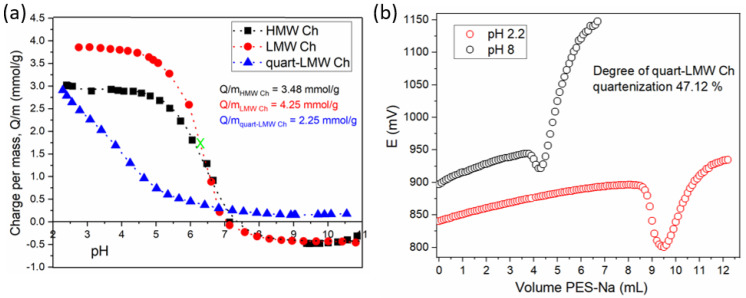
(**a**) Potentiometric titration curve expressed as a charge per mass for three types of Chs and (**b**) the polyelectrolyte curve at pH 2.2 and 8 for quart-LMW Ch.

The IR spectra for all three types of Chs are shown in Figure 1a,b. LMW and HMW Chs have similar positions of the bands of the functional groups, typical of their structures, differing only by MW and the degree of deacetylation (Scheme 1). The broad band at approximately 3400 cm^−1^ corresponds to –OH and –NH vibrations and inter- and intramolecular hydrogen bonds formed between the Ch polymer molecules. In agreement with the polymer structure, the band at 2900 cm^−1^ is attributed to C–H stretching, at 1654 cm^−1^ to –CO–NH– (amide I) stretching, and at 1581 cm^−1^ to N–H bending. The intense peak at approximately 1050 cm^−1^ is attributed to glycoside bonds (i.e., –C–O–C vibrations) in all polymers. In the case of quart-LMW Ch, the spectrum differs from LMW Ch at wavenumbers typical of trimethylammonium groups at 3100–3020 cm^−1^ (magnified part of the spectrum with a prominent band at 3026 cm^−1^ in Figure 2b) and has a pronounced new peak at 1485 cm^−1^, and a band at 960 cm^−1^. The latter clearly indicates a successful derivatization of the primary amino group of LMW Ch to quaternary ammonium groups of quart-LMW Ch. This is indeed observed by the gradual decrease in the peak, typical of N–H bending (at 1581 cm^−1^), and the appearance of the new band at 1485 cm^−1^, i.e., (CH_3_)_3_N^+^ [42,43].

#### 3.1.2. X-ray Photoelectron Spectroscopy (XPS)

Characteristic elements such as carbon, oxygen, and nitrogen are found in the HMW and LMW Ch samples, and in its quart-LMW Ch derivative (Table 1, Figure 1c). Some chlorine also appears in the quart-LMW Ch sample, likely originating from the GOPTS reagent that is needed in the modification process (Scheme 1). For all three Chs, there is no significant difference in the high-resolution O 1s spectrum (Figure 1d), while in the C 1s carbon spectrum, the fraction of C–C bonds is larger than the C–O fraction in the quart-LMW Ch and HMW Ch sample, and C–O bonds predominate in the LMW Ch sample (Figure 1e). The largest difference is observed in the N 1s nitrogen spectrum (Figure 1f), which shows two peaks in the quart-LMW Ch sample. The new peak, appearing at a higher energy (~402.4 eV) in quart-LMW Ch, belongs to quaternary nitrogen, while the peak characteristic of amino groups (–NH_2_) appears at a binding energy of 399.2 eV for HMW and LMW Ch (Figure 1f). The latter is again clear evidence of successful LMW Ch derivatization.

#### 3.1.3. Potentiometric and Polyelectrolyte Titration

The HMW and LMW Chs protonate/deprotonate in a 1-p*K* manner (Figure 2a) with a similar p*K* value of approximately 6.4 (marked with a green sign in Figure 2a). However, the difference is in the total charge per mass, which is 4.25 mmol/g for LMW Ch and corresponds to 3.9 mmol/g to cationic groups, while a slightly lower value was obtained for HMW Ch (3.5. mmol/g, of which 3 mmol/g belongs to cationic groups). It is also evident from the behaviour of the potentiometric curve that LMW and HMW Chs exhibit a negative charge in the alkaline range. A negative charge in the alkaline range is due to some residual deprotonated acetyl groups. On the other hand, in the acidic region, both show a positive charge due to protonated primary amino groups (i.e., –NH_3_^+^). The successful derivatization is also confirmed by the different charge behaviour of quart-LMW Ch, which deprotonates and reaches a plateau value of approximately 0.25 mmol/g as it approaches the alkaline region (Figure 2a). The latter was further supported by polyelectrolyte titration for quart-LMW Ch at two different pH values (Figure 2b), where at pH 2.2 both amino (primary and quaternary) groups are protonated, while at pH 8 only quaternized amino groups have a positive charge. This is in accordance with isoelectric points determined by potentiometric titration, which was clearly seen in both primary Chs, for which full deprotonation occurred above pH 7–7.5. Polyelectrolyte titration by both chosen pHs allowed the determination of the degree of quaternization, which was approximately 47%, while the other 53% corresponded to unmodified –NH_2_ groups.

### 3.2. Antiviral Activity of Bioactive Chitosans against Phi6

Antiviral activity, i.e., inactivation potential of LMW and HMW Chs (Table 2) against phi6, is high at low pH (4.5 ± 0.3), averaging 6.77 log for LMW Ch. This results in 100% phi6 inactivation, while it is slightly lower for HMW Ch, averaging 6.32 log or 99.9995%. At the same pH, a significantly lower antiviral activity was observed for quart-LMW Ch, which accounted for 0.67 log or 78.76% inactivation. To exclude the influence of concentration, all agents were tested at the same mass concentration of 1.25 mg/mL. Conversely, a completely different behaviour was detected in the neutral pH, i.e., at 7.4, where both pristine Chs show no inactivation (Table 2), while derivate quart-LMW Ch shows inactivation of 1.55 log (Table 2). This can be explained in terms of absence or a significantly lower total accessible positive charge in the case of both LMW and HMW Ch, as revealed by the potentiometric titration curve (Figure 2a). Quaternary amino groups in derivate quart-LMW, however, carry a permanent positive charge regardless of pH value (Scheme 1, Figure 1 and Figure 2).

In order to achieve greater insight into the mechanisms involved in the interaction between phi6 and Chs, additional properties were examined, including morphology, elemental composition, particle size, and zeta potential measurements.

### 3.3. Interactions between Phi6 and Chitosans, and Mechanism of Inactivation

**Figure 3:**
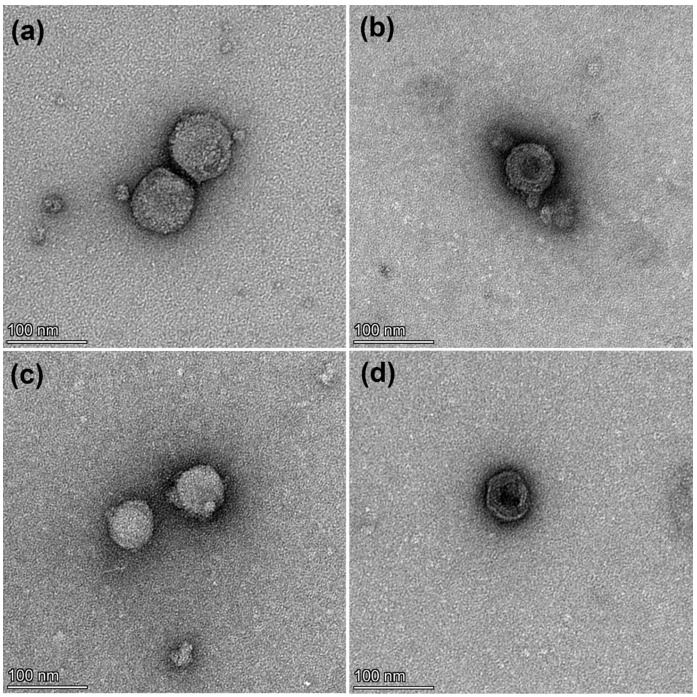
Representative micrographs of bacteriophages phi6. (**a**) Bacteriophages of positive control, i.e., sample without Chs, were covered with envelope. (**b**) Damaged bacteriophage phi6 after incubation with LMW Ch. (**c**) Bacteriophage without envelope after incubation with HMW Ch. (**d**) Damaged bacteriophage phi6 after incubation with HMW Ch.

**Figure 4:**
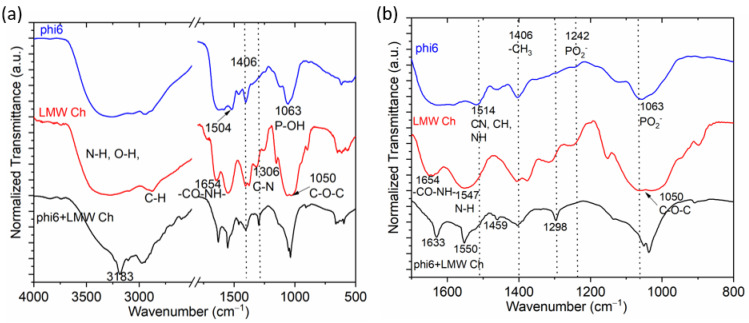
(**a**) ATR-FTIR spectra of phi6 and LMW Ch alone, and their combination after 2 h of incubation and (**b**) smaller inset with the most important bands marked.

A TEM analysis was used to visualize the morphology of phi6 alone (Figure 3a) and in combination with Chs (Figure 3b–d, Figure S1 in Supplementary Material). Contact of viruses with Chs resulted in a lower number of particles observed on the micrographs (especially after incubation with LMW Ch, data not shown) and in different stages of damage that the particles expressed (Figure 3b–d). The damaged viruses were smaller due to the absence of the lipid envelope, the remnants of which can be seen around the viruses (Figure 4b,c). The size of phi6 after contact with Ch was approximately 63 nm (60–65 nm), instead of 85 nm (81–89 nm), which was the diameter displayed by viruses in positive controls (Figure 3a). Since phi6 (among others) requires an intact lipid layer to successfully infect bacteria, the change in virus morphology (especially the complete loss of the lipid layer) affects its infectivity. A similar observation has previously been made by [44] when the interaction between bacteriophage phi6 and montmorillonite clay was studied using TEM. It was observed that phi6 swells and disassembles after contact with the positively charged platelet edges of the clay, resulting in partial or even complete loss of the envelope. In the case of bacteriophage T2, significant structural changes were observed after contact with Ch, such as the absence of long-tailed fibers and a deformed basal plate [45]. Moreover, Ch can also directly affect the receptor-recognizing structures of bacteriophages [23].

The possible interactions for exemplary chosen LMW Ch and phi6 after 2 h of incubation were attempted to be additionally explained by position changes of the spectral bands, observed by infrared spectroscopy (Figure 4). For bacteriophage phi6 (spectra marked in blue), the main bands corresponding to the phospholipid envelope were identified. These are vibrations for C–H, –CH_2_, –CH_3_, and C–O in hydrocarbon chains, at 1050 cm^−1^ C–O–C in head groups, most likely originating from phosphatidylethanolamine and phosphatidylglycerol [46,47,48,49,50]. Signals for phosphate units were assigned at 1242 and 1063 cm^−1^ for PO_2_^−^. These are associated not only with lipids, but also with bacteriophage phi6 proteins. Strong signals attributable to capsid proteins were also obtained, including –CH_3_ (2871 cm^−1^) from threonine, alanine, valine, leucine, methionine, amide I (1654 cm^−1^), –CN, –CH, and –NH of tryptophan (1514 cm^−1^) (spike protein P3, fusion protein P6, capsid protein P1), and -CH_3_ at 1457 and 1406 cm^−1^ [46,47,48,49,50]. However, it should be noted that spectral interpretation of complex biological structures, such as bacteriophage phi6, is rather suggestive [48]. After incubation and interaction of phi6 with LWM Ch, changes in the spectrum can be observed (Figure 4b). In particular, changes in the position of the phosphate groups (or their absence), and changes in the band shapes at higher wavenumbers typical of amino groups of LMW Ch (at 1547 cm^−1^ and approximately 3300 cm^−1^ band of phi6 + LMW Ch changes shape and intensity, and shifts were observed for N–H vibrations), indicate changes in the elemental structure after contact between the two components. The results are consistent with the prediction of electrostatic attraction between the phospholipid envelope and the positively charged LMW Ch.

The zeta potential (ZP; Table S3 in Supplementary Material) and the hydrodynamic diameter (HD; Table 3) of the individual suspension of components (i.e., LMW, HMW, and quart-LMW Chs, and phi6), and also of the combination of each Ch with phi6, were determined. Samples were prepared in the same way as described in Section 2.5.1. According to literature data, bacteriophage phi6 shows IEP at pH 7 [51]. The ZP of the latter showed that it is negatively charged at pH 7.4, while it has a low positive ZP in the slightly acidic range. The data are consistent with HD, where larger particles are expected near IEP, while repulsive forces are present at lower pH values, resulting in smaller bacteriophage size. Generally, ZP is strongly influenced by pH and also by ionic strength [41]. At this point, it should also be emphasized that the 1 × SM buffer contains a range of ions (i.e., Na^+^, Cl^−^, Mg^2+^, SO_4_^2−^, etc.), and ensures an ionic strength that can have an impact on the aggregation and charging behaviour of all components (the thickness of the electrostatic double layer decreases). The ZP results of the individual Chs and those in combination with phi6 at two different pH values show negligible differences in ZP after contact with phi6 (Table S3). This may suggest that this parameter is not valuable enough to follow the possible mechanism of inhibition.

**Figure 5:**
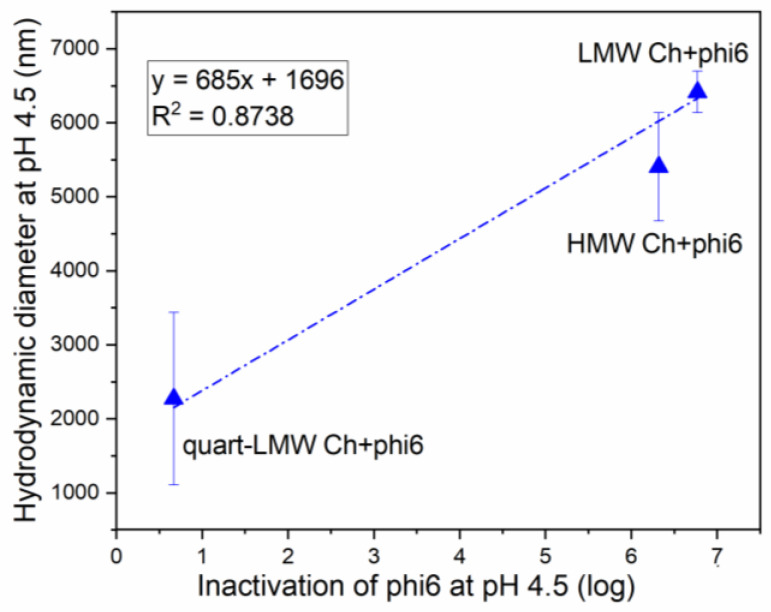
Correlation between inactivation activity of bacteriophage phi6 at pH 4.5 and hydrodynamic diameter after incubation of phi6 + Chs antiviral agents.

In contrast to ZP, some conclusions can be drawn from the HD measurements at pH 4.5, where larger HDs were observed after contact between Chs and phi6 than for each individual component (Figure S2a–c in Supplementary Material), most likely due to Coulombic interactions between Chs and phi6, causing damage to the viral outer layer. Similar observations were made with TEM (Figure 3) and the ATR-FTIR (Figure 4), and it is consistent with the observations of Milewska et al., who found that cationic components react with coronaviruses by electrostatic attractive forces [36]. Moreover, Chs may interact not only chemically, but also physically, as some amino groups are already deprotonated at a measured pH. Phi6 may also form aggregates below its IEP (Table 3), as already demonstrated for bacteriophage MS2 [52]. Therefore, some contribution from the interactions between the components themselves should also be considered. The results of HD correlate well with virus inactivation results (Table 3). The largest size distribution is observed in the case of LMW Ch + phi6, where the highest inactivation activity was also observed, similar to HMW Ch + phi6. In the case of quart-LMW Ch incubated with phi6, a larger HD was observed, but to a lesser extent than the other two Chs. The dependency between HD and inactivation at pH 4.5 also appears to have a good linear relationship, with a correlation coefficient of R^2^ = 0.8738 (Figure 5). However, this is only a rough estimation, due to the fact that only tree points may be taken into account.

It can be concluded from Figure 5 that a higher inactivation activity is connected to higher HD, meaning that more expressive electrostatic attractive interactions between both components (i.e., Ch and virus) lead to phi6-Ch interactions. For instance, in the case of LMW Ch and phi6, the average HD increased by 302% in comparison to LMW Ch alone (Table 3). This was due to the interaction between both components, leading to a larger size. This was also confirmed by larger polydispersity index (PDI) values (more heterogeneous size distribution) compared to the PDI values of the pure Chs agents (Table 3). At a neutral pH after contact between the Chs and phi6, the size distribution of the Chs did not change significantly, and even decreased in the case of LMW Ch and HMW Ch in comparison to HD at pH 4.5 (Figure S2d,e in Supplementary Material; Table 3). Nevertheless, a larger HD was observed at pH 7.4 for quart-LMW Ch (Figure S2f in Supplementary Material), which also showed high antiviral activity at this pH (Table 2).

That electrostatic interactions are the driving force behind the inhibition of phi6 is evident from the results of the infectivity tests, which clearly indicate a dependence of phi6 inactivation on pH, and consequently on the corresponding charge of the antiviral agent (Figure 2a, Table 2).

**Figure 6:**
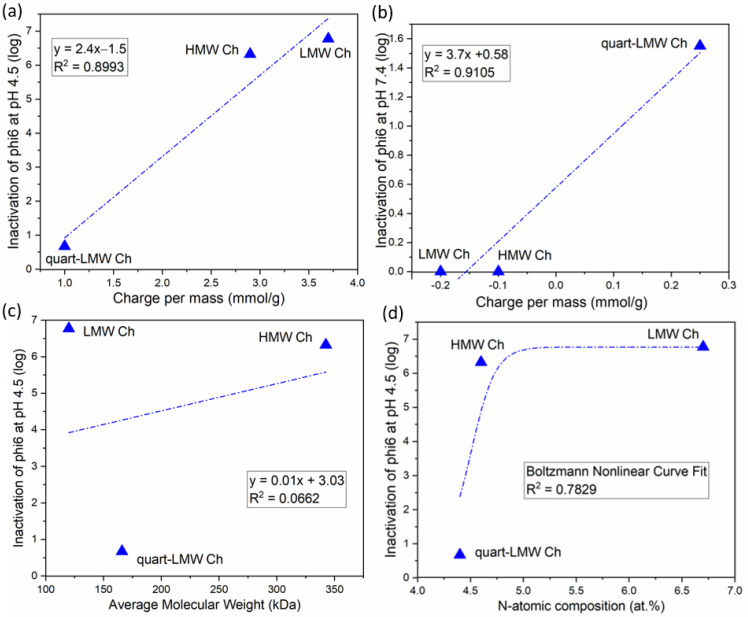
(**a**) correlation between total Ch charge and inactivation of bacteriophage phi6 at pH 4.5 and (**b**) pH 7.4, (**c**) the inactivation of phi6 at pH 4.5 towards the average MW of different Chs, and (**d**) relation between N-atomic concentration and inactivation of phi6 at pH 4.5.

In the case of the branched structure of the quaternary amino groups in quart-LMW Ch, steric forces may also be involved in the inactivation mechanism. The correlation between surface charge and antiviral activity at both pH 4.5 and 7.4 showed a large linear correlation with coefficients of R^2^ = 0.8993 and R^2^ = 0.9105, respectively (Figure 6a,b). Generally, a coefficient greater than 0.7 is considered a strong correlation [53]. This is in agreement with the results of Kochikina and Chirknov [25,26], who showed that anionic chitosan derivatives did not cause inactivation, while cationic ones were efficient, demonstrating the importance of the polycationic nature of Ch molecules for bacteriophage inactivation. However, in the same studies, no linear correlation was found between the number of amino groups and inhibition of T2 and T7 coliphage infection. 

Moreover, the positive charge of Chs is directly related to the degree of deacetylation and (Scheme 1) closely linked to MW. LMW Ch and synthesized quart-LMW Ch exhibit a higher degree of deacetylation than HMW Ch (see Scheme 1), but in our work there was no significant difference in their activity against phi6. The latter is in line with the findings of Davydova et al.; their research indicated that the antiviral activity of Chs was almost unaffected, even by the degree of deacetylation [31]. It was also suggested that MW has an impact on antiviral activity, which decreases with increasing MW [31]. This was not found in our research, and no particular dependence was found between the average MW and inactivation of phi6 (pH 4.5, Figure 6c). The atomic nitrogen composition and its relationship with the inactivation activity of phi6 showed no particular correlation, as the inactivation activity was significantly different for similar N-atomic compositions of quart-LMW Ch and HMW Ch (Figure 6d). From this, it can be seen that the protonation of the amino group, and thus the positive charge, is crucial for inactivation of enveloped phi6.

Thus, clearer mechanisms of the interactions can be interpreted by the fact that bacteriophage phi6 has a phospholipid shell [46,54]. Due to the cationic nature of LMW Ch and HMW Ch under IEP (Figure 2a), the mechanism for the inhibition of phi6 could be ionic interactions between negatively charged shells with protonated amino groups. Physical and hydrophobic interactions could also be simultaneously involved. It has been shown previously that the phospholipid bilayer of bacteriophage phi6 is composed of P9 protein, and its amino acid composition has up to 11 mol% glutamic acid [55]. This suggests that a negative charge is predominant in the structure of the outermost layer of the particle, and that the bacteriophage envelope dictates how the components react and affect aggregation, viral stability, and infectivity [44].

For agents based on quaternary ammonium, virucidal activity has been explained by binding them to nucleic acid, or by degradation of virions through interaction with lipids or proteins [56]. This may also be true for inactivation of phi6 by quart-LMW Ch. The report by Hutchison et al. [57] determining the resistance profile of bacteriophage phi6 to quaternary ammonium compounds under a variety of test conditions is supported by our observations of lower inactivation activity of quart-LMW Ch.

**Scheme 2:**
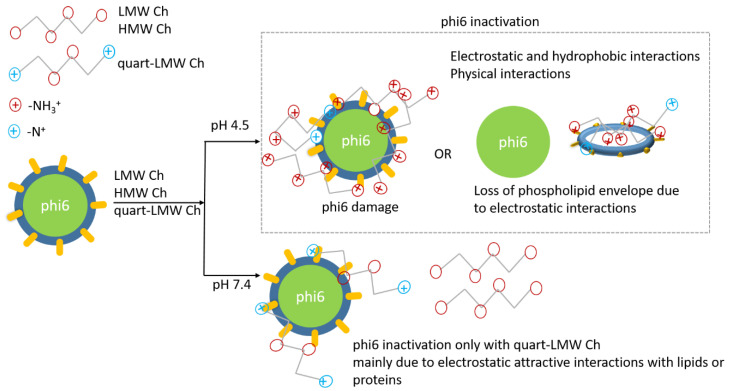
Proposed inhibitory mechanism between Ch antiviral agents and bacteriophage phi6, a surrogate for SARS-CoV-2.

A proposed schematic presentation of possible phi6 inhibitory mechanisms is illustrated in Scheme 2. These predictions were indeed confirmed by the TEM analysis (Figure 3), which revealed the absence of the envelope. Similar results were observed by Alhakamy et al. [58].

It is important to understand the influence of physicochemical properties of Chs in model viruses. These findings are important and can be applied to a broader spectrum of pathogenic viruses, such as SARS-CoV-2. In addition, an in-depth look at the interaction between Chs and phi6 is very important for future use of Chs as a coating. It is clear that the coating must be applied to solid materials under conditions that allow a large availability of amino groups on the material surface.

## 4. Conclusions

In conclusion, the inactivation potential of chitosan-based polymers against bacteriophage phi6, a widely recognized surrogate for SARS-CoV-2, was demonstrated, and the mechanism behind it was investigated. First, characteristic elements and functional groups in HMW Ch, LMW Ch, and its quart-LMW Ch derivative were confirmed. Thus, the successful derivation of LMW Ch to quart-LMW Ch was demonstrated, and the degree of quaternization determined by titrations was approximately 47%. In addition, the titration techniques provided the number of positive charges in each product in the following order for the highest amount of primary amino groups: LMW Ch, HMW Ch, and quart-LMW Ch derivative. The inactivation ability of Chs was dependent on pH, with a strong (>6 log) reduction at a low pH in LMW and HMW Chs, and a fairly high reduction in quart-LMW Ch at a neutral pH. A TEM analysis showed the damage to virus phi6 after incubation with Ch, proving the disintegration of viral structure. In addition, the ATR-FTIR technique also indicates the interactions occur due to changes in the bands, typical of chitosan or the viruses themselves. Dynamic and electrophoretic light scattering studies with ZP and HD were also performed, and it was concluded that higher inactivation activity consequently led to a higher HD, as a consequence of the more pronounced attractive interactions between both components leading to their interactions. The results suggest that electrostatic interactions are the main driving forces for the action of chitosans as antiviral agents on phi6. This knowledge is extremely important for the further use of antiviral chitosans as coatings.

Although chitosan-based agents have demonstrated antiviral activity against various bacteriophages and viruses [26,27,28,30,31,32], the inactivation potential against phi6 has not yet been investigated, highlighting the originality of this work and the important addition to research on the antiviral activity of chitosan. In the case of SARS-CoV-2 virus, much attention has been paid to the development of chitosan-based vaccines and drugs [11,12,14], but not to the protection of PPE in the form of bioactive polymer surface coatings to prevent cross-infection or transmission of enveloped viruses, such as SARS-CoV-2. Therefore, this study presents the fundamentals of the behaviour of phi6 and chitosans, which are also relevant to SARS-CoV-2.

When bioactive coating is applied to a solid material, a complex and heterogeneous structure is formed, and understanding the mechanism of the antiviral action of chitosan itself is essential for manipulating the coating conditions to maximise the efficacy of the antiviral chitosan activity on the material surface.

## Figures and Tables

**Table 1:** Atomic elemental composition of different Chs in atomic % (at.%).

Antiviral Agent	Elemental Composition (at.%)					
	C	N	O	Si	Cl	Ca
HMW Ch	70.6	4.6	23.8	0.7	/	0.4
LMW Ch	63.0	6.7	30.3	/	/	/
quart-LMW Ch	72.4	4.8	20.1	1.2	1.5	/

**Table 2:** Inactivation of bacteriophage phi6.

Antiviral Agent	pH	Inactivation			
		Log	Average (log)	%	Average (%)
LMW Ch	4.5 ± 0.3	6.80	6.77	100.0	100.0
		6.74		100.0	
	7.4 ± 0.4	0	0	0	0
Quart-LMW Ch	4.5 ± 0.3	0.67	0.67	78.76	78.76
	7.4 ± 0.4	1.60	1.55	97.48	97.15
		1.59		97.42	
		1.46		96.56	
HMW Ch	4.5 ± 0.3	6.19	6.32	99.99994	99.99995
		6.45		99.99996	
	7.4 ± 0.4	0	0	0	0

**Table 3:** Results of average hydrodynamic diameter (HD) (3 measurements) of individual and merged components in the 1 × SM buffer. The last column represents the increased/decreased ratio of HD with respect to a specific chitosan.

Model Virus (10^6^ PFU/mL)	Antiviral Agent (1.25 mg/mL)	pH in One Point	Transmittance (%)	HD-Average (nm)	PDI (%)	Increased HD with Respect to Ch (%)
phi6	/	7.4 + 0.4	89	3874 ± 1343	36	/
phi6	/	4.5 ± 0.3	89	600 ± 23	25	/
/	quart-LMW Ch	4.5 ± 0.3	89	1250 ± 163	21	/
/	HMW Ch		90	964 ± 371	27	/
/	LMW Ch		89	1598 ± 548	31	/
/	quart-LMW Ch	7.4 + 0.4	85	1577 ± 412	28	/
/	HMW Ch		87	1573 ± 415	34	/
/	LMW Ch		84	8380 ± 1439	39	/
phi6	quart-LMW Ch	4.5 ± 0.3	80	2271 ± 1164	36	82
phi6	HMW Ch		88	5405 ± 730	48	461
phi6	LMW Ch		86	6417 ± 282	30	302
phi6	quart-LMW Ch	7.4 + 0.4	78	2207 ± 296	37	77
phi6	HMW Ch		83	1787 ± 660	39	85
phi6	LMW Ch		86	2751 ± 88	29	72

## Data Availability

The data presented in this study are available upon request from the corresponding author.

## Supplementary Materials

The following supporting information can be downloaded at: https://www.mdpi.com/article/10.3390/polym14163357/s1. Figure S1: representative damaged phi6 after interaction with quart-LMW Ch. Figure S2: exemplary shown hydrodynamic diameter for HMW, LMW Ch, and quart-LMW Ch at pH 4.5 (a,b,c), and at pH 7.4 (d,e,f) expressed as intensity distribution data. Table S1: minimum inhibitory concentration (MIC) for bacteria. Table S2: determination of minimum inhibitory concentration for *P. syringae*. Table S3: results of average ZP (three measurements) of individual and merged components in the 1 × SM buffer.
