# Supplementary material for: First Insights into the Antiviral Activity of Chitosan-Based Bioactive Polymers towards the Bacteriophage Phi6: Physicochemical Characterization, Inactivation Potential, and Inhibitory Mechanisms
Source: Polymers (Basel). 2022 Aug 17;14(16):3357. doi: 10.3390/polym14163357 (PMC9413598; doi:10.3390/polym14163357)
Supplement: Supplementary file 1 [file polymers-14-03357-s001.zip › polymers-1850431-supplementary.pdf]

Supplementary Materials

1. Minimum inhibitory concentration (MIC) of the test agents on bacteria

Minimum inhibitory concentration (MIC) of the test agents, e.g., LMW Ch, quart-LMW Ch, or HMW Ch, was determined in liquid medium for *Pseudomonas syringae* (Tables S1,2).

Table S1. Minimum inhibitory concentration (MIC) for bacteria. The pH values represent the values of the test agents prior to the addition of the bacterial culture.

Test agent	MIC
	P. syringae
LMW Ch (10 mg/L), pH = 4	1:80
Quart-LMW Ch (5 mg/L), pH = 5	> 1:20
HMW Ch (10 mg/L), pH = 4	1:20

Table S2. Determination of minimum inhibitory concentration for *P.syringae*; ++ good bacterial growth; + partial inhibition of bacterial growth; +/− strong inhibition of bacteria growth, − no bacterial growth.

**Sample Dilution**			**Undiluted**	**1:2**	**1:4**	**1:8**	**1:16**	**1:32**
**Final Dilution in the Test Tube**			**1:20**	**1:40**	**1:80**	**1:160**	**1:320**	**1:640**
Test agent	LMW Ch	Bacterial growth	−	−	−	+	++	++
	quart-LMW Ch		++	++	++	++	++	++
	HMW Ch		−	−/+	+	++	++	++

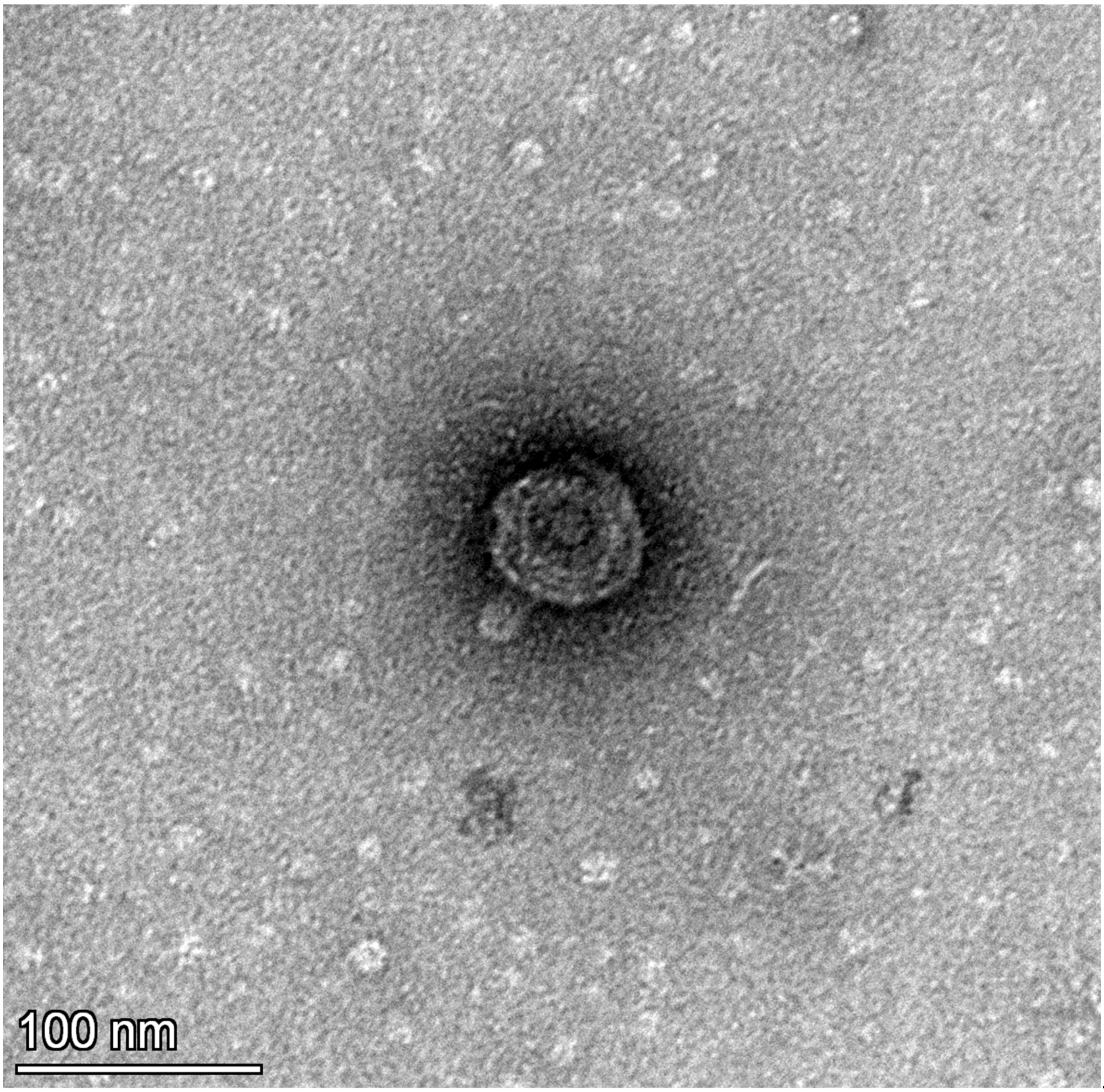

Figure S1. Representative damaged phi6 after interaction with quart-LMW Ch.

Table S3. Results of average ZP (3 measurements) of individual and merged components in 1 × SM buffer.

Model Virus/ 10^6 PFU/mL	Antiviral Agent at Concentration 1.25 mg/mL	pH in One Point	Transmittance (%)	ZP-Average (mV)
phi6	/	7.4 + 0.4	89	−2.7 ± 4.1
phi6	/	4.5 ± 0.3	89	0.8 ± 1.2
/	quart-LMW Ch	4.5 ± 0.3	89	3.6 ± 0.9
/	HMW Ch		90	14.7 ± 0.9
/	LMW Ch		89	18.7 ± 1.8
/	quart-LMW Ch	7.4 + 0.4	85	2.6 ± 0.3
/	HMW Ch		87	6.5 ± 0.5
/	LMW Ch		84	7.3 ± 0.3
phi6	quart-LMW Ch	4.5 ± 0.3	80	2.7 ± 0.3
phi6	HMW Ch		88	17.8 ± 1.3
phi6	LMW Ch		86	17.7 ± 0.3
phi6	quart-LMW Ch	7.4 + 0.4	78	5.1 ± 0.1
phi6	HMW Ch		83	7.6 ± 0.1
phi6	LMW Ch		86	6.0 ± 0.2

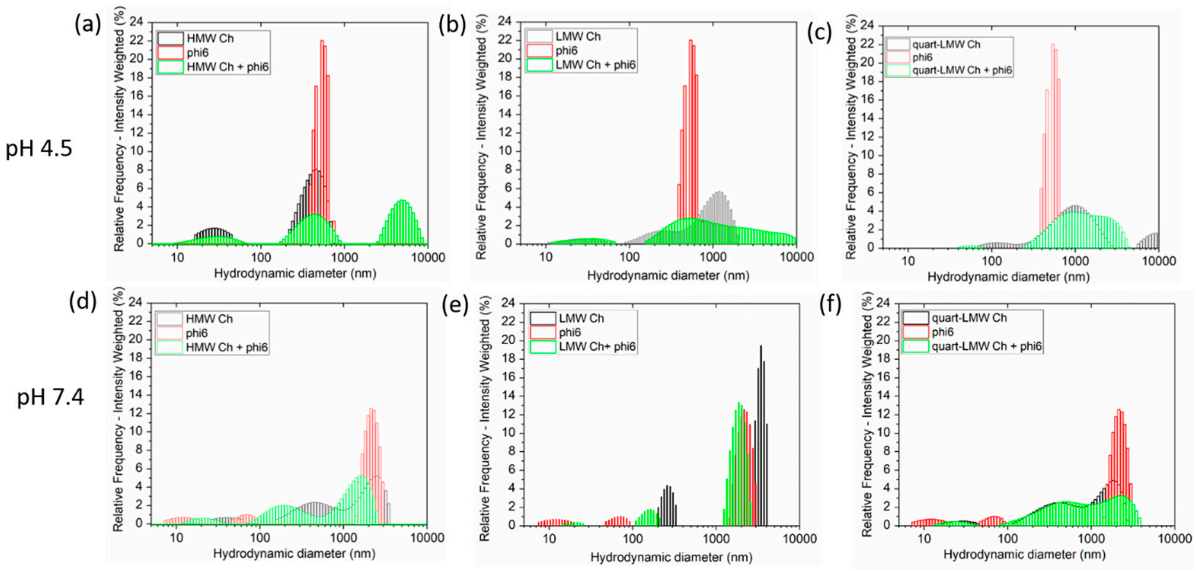

Figure S2. Exemplary shown hydrodynamic diameter for HMW, LMW Ch and quart-LMW Ch at pH 4.5 (**a,b,c**), and at pH 7.4 (**d,e,f**) expressed as intensity distribution data.
